# An Expert Knowledge Algorithm and Model Predicting Wound Healing Trends for a Decision Support System for Pressure Injury Management in Home Care Nursing: Development and Validation Study

**DOI:** 10.2196/65716

**Published:** 2025-12-09

**Authors:** Aya Kitamura, Aruto Ando, Gojiro Nakagami, Hiromi Sanada

**Affiliations:** 1 Department of Gerontological Nursing, Ishikawa Prefectural Nursing University, Ishikawa, Japan; 2 Department of Gerontological Nursing and Wound Care Management, Graduate School of Medicine, The University of Tokyo, Tokyo, Japan; 3 Ishikawa Prefectural Nursing University, Gakuendai 1-1, Kahoku-city, 929-1210, Japan, 81 76-281-8400

**Keywords:** algorithm, care recommendation, clinical decision support system, home care, pressure injury

## Abstract

**Background:**

Home-visiting nurses have difficulty selecting appropriate pressure injury (PI) management despite using clinical practice guidelines in various home-visiting settings. Clinical decision support systems can help home-visiting nurses’ decision-making.

**Objective:**

This study aimed to develop a care algorithm reflecting the expertise of a wound expert nurse and a predictive model for the change of PI severity to inform home-visiting nurses to receive actual consultation.

**Methods:**

First, an existing algorithm was modified by semistructured interviews with a certified wound expert nurse. Case information was input into both base and high-expertise algorithms, which provided care recommendations across 9 fields: (1) pressure relief; (2) nutritional management; (3) shear relief; (4) moisture management; (5) wound dressing use; (6) care for physical factors including bone prominence, obesity, joint contractures, and periwound edema; (7) care for systemic disorder; (8) selection of wound dressings, ointments, and negative pressure wound therapy; and (9) wound cleansing. An expert interviewee assessed the high-expertise algorithm’s recommendations on a 5-point scale, comparing them to the base algorithm and their own clinical judgment. To measure the algorithm’s applicability, agreement proportions were calculated as the number of vignettes where the care recommendation was considered appropriate or total number of vignettes. To measure the algorithm’s alignment, improvement proportions were calculated as the number of vignettes where the care recommendation improved or total number of vignettes excluding vignettes when the existing and high-expertise algorithm both showed an appropriate recommendation. Expected healing levels were evaluated by a 4-point scale where 4 indicates the high-expertise algorithm can “much improve” the case. Second, predictive distributions of changes in DESIGN-R 2020 score, PI severity score, were estimated with a hierarchical Bayesian model. The best model determined using training data (n=42) calculated coverage probabilities of 90% prediction interval in test data (n=34). The coverage probability of a 90% prediction interval was defined as follows: the number of times when actual scores were within the 90% prediction interval or the number of assessments when the prediction was conducted.

**Results:**

The agreement proportions were 0.92 (33/36), 0.75 (27/36), and 0.89 (32/36) for each round. The improvement proportions were 0.73 (8/11), 0.25 (3/12), and 0.76 (13/17), respectively. The expected healing level was 2.67, 3.00, and 3.25, respectively. Coverage probabilities of 90% prediction interval in the test data were 0.67 (4/6), 0.83 (5/6), 0.86 (6/7), and 0.80 (8/10), respectively.

**Conclusions:**

This study developed an algorithm reflecting the expertise and a model to estimate predictive distributions of changes of DESIGN-R 2020 score for developing clinically applicable clinical decision support systems for home-visiting nurses providing appropriate PI management.

## Introduction

Pressure injury (PI) is a localized damage to either the skin or the underlying tissue, or to both, caused by pressure and shear [1] and has various negative impacts on patients and the health care economy [2,3]. Although prevention is the most important for PIs, nurses must select the most appropriate management for early healing when a PI occurs. As home health care has become highly developed, home-visiting nurses are increasingly faced with managing more severe PIs. In hospitals, PIs are managed without worsening because of the standardized care environment, the presence of wound specialists, and the appropriate application of clinical practice guidelines. On the other hand, in home care settings, the care environment is diverse, and accessibility to specialists is limited, making it difficult to implement the standardized care recommended by the guidelines. There, it is difficult to heal severe PIs at home. In fact, the proportion of severe PIs extending beyond the dermis was 38.8% in home-visiting settings compared with 20.1% in university hospitals in Japan [4]. As a result, home-visiting nurses who are not specialized in PI management have difficulty determining appropriate care because clinical guidelines of PI management do not provide a specific recommendation to each case [1,5]. In this situation, there is a high need for consultation with specialists. Sood et al reported that preexisting wound treatment was altered in 64% of cases when home-visiting nurses consulted wound experts [6]. However, the number of experts who can provide knowledge is not always sufficient in some countries and regions. While there are more than 2500 certified wound, ostomy, and continence (WOC) nurses in Japan, approximately 90% belong to hospitals, especially those with many beds [7]. The fact that the utilization proportion of the public payment system, in which a WOC nurse visits a PI patient’s home together with a home-visiting nurse, is <5% indicates the difficulty of home-visiting nurses to undergo consultation from a WOC nurse [8]. Although telemedicine or telenursing can improve accessibility for expertise [9] and patient outcomes [6,10], the high prevalence of PIs in home care settings, ranging from 3.3% to 11.1% [11], makes it difficult to provide consultation for all cases requiring specialized management.

This study focused on a clinical decision support system (CDSS) to address this challenge. A CDSS provides assessment and care recommendations based on individual patient characteristics to support decision-making [12]. Although few studies have reported the effect of the CDSS on PI management [13,14], it could help determine care strategies, especially for less experienced nurses [14].

Knowledge-based CDSSs are designed based on clinical practice guidelines and consolidated expert opinions. Evidence is essential, but its coverage is narrow. Expert opinions also lose flexibility in the consensus method. Therefore, existing CDSS recommendations are often immature and unacceptable to nurses, especially in diverse home-visiting settings [15]. In contrast, expertise covers not only knowledge from previous studies but also considerations for clinical situations and patient preferences [16]. It indicates that reflecting an expert nurse’s expertise to an existing care algorithm may make the CDSS mature and clinically applicable. However, such a CDSS has not been developed.

Additionally, a CDSS needs to inform home-visiting nurses that consultation from an expert nurse is needed when the CDSS algorithm cannot address the patient’s management appropriately as a fail-safe mechanism. The appropriateness of the CDSS for the patient is indicated by comparing the actual healing progress of the wound with the expected healing progress under expert consultations. While many previous studies have predicted the healing trajectory of PIs by point estimation [17-20], distributional estimation is more informative in determining how much worse the wound is than predicted. Xu et al [21] modeled the healing trajectory using a generalized linear mixed model but did not use information that experts generally consider, such as wound location, status, or the patient’s general condition. A review of previous studies found no studies that used PI location or patient characteristics to estimate the predictive distribution of PI healing.

The goal is to develop a CDSS with 2 features: (1) a care selection algorithm that reflects the expertise of PI experts and (2) a fail-safe system that informs nurses whether they should receive an actual consultation. As the first step, we have conducted 2 studies: the development of a care algorithm that reflects the expertise of a wound expert nurse (Study I) and a predictive model that estimates the predictive distribution of the changes in PI severity score (DESIGN-R 2020) [22,23] with patients’ information (Study II).

## Methods

### Study I

#### Outline

First, a base care algorithm and an optimal expert were selected to develop an algorithm reflecting PI management expertise. Next, semistructured interviews were conducted with the expert nurse about the algorithm’s content. During the interviews, the interviewee was asked to review the base algorithm. The researcher (AA) modified it to make a high-expertise algorithm based on the interviewee’s comments. Finally, the researcher (AA) verified that the high-expertise algorithm reflected the expertise of the interviewees better than the base algorithm. This process was conducted in 3 rounds using PI clinical vignettes. The algorithm was modified based on the interviewee’s feedback at the end of each round.

This algorithm was developed for PI care of nonterminally ill older patients at home to promote wound healing. Since the pathophysiology of PIs in the lower extremities is more complicated than that in other parts of the body, PIs in the lower extremities were excluded from this study.

The base and high-expertise algorithms were implemented as a system that automatically outputs care recommendations in response to the input information with Python 3.9.7 (Python Software Foundation, USA).

#### A Base Algorithm

As if-then rules and flowcharts are easy for health care professionals to understand and reflect expertise intuitively, we adopted an algorithm composed of these as the base algorithm. Among several care algorithms for PI management using flowcharts [24-26], this study selected the algorithm developed by Sanada [24] as a base algorithm. We selected this algorithm because it extensively describes nursing skills to be performed based on the causes of PI development and delayed wound healing.

**Figure 1.**
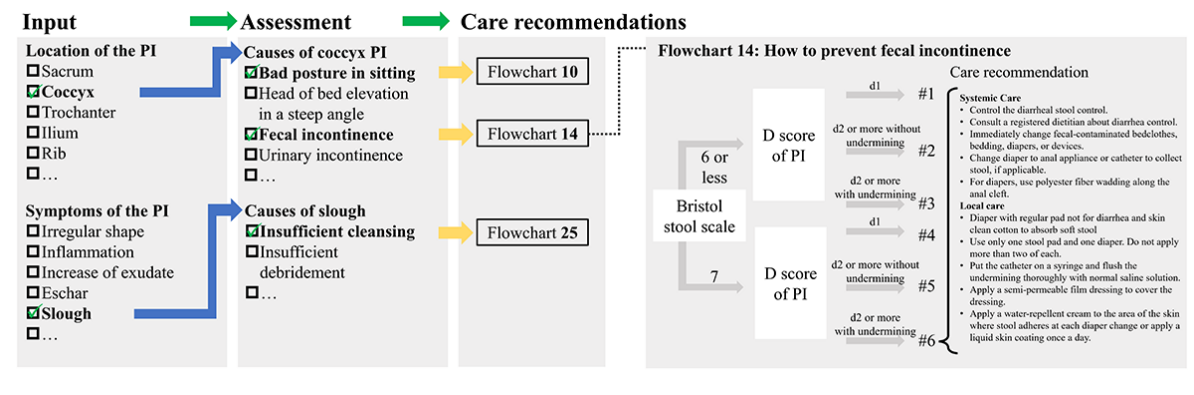
Care algorithm for pressure injury. PI: pressure injury.

The base algorithm was developed with the evidence and multiple expert opinions obtained by a consensus method in 2003 [27]. The algorithm improved PI healing compared to the control group in a hospital in an observational study, and after that, it was modified by applying to actual cases [27]. As shown in Figure 1, the algorithm consists of lists of PI locations and symptoms, sublists of causes of PI occurrence or delayed healing, and care flowcharts corresponding to the items in the sublists. When a nurse inputs information about the location and symptoms of the patient’s PI into the algorithm, a corresponding sublist of causes of PI occurrence or delayed healing and flowcharts to select appropriate care are proposed to the user.

#### Interviewee and Interviewer

The base algorithm was updated and modified to fit the situation of the current PI management in home care settings by interviews with a WOC nurse. A WOC nurse with a high level of expertise in PI management was recruited as an interviewee. The interviewee had >30 years of clinical experience and was capable of providing consultation on hard-to-heal PI management at home care settings in a previous study [9]. The interviewer attended approximately 10 PI consultations with the interviewee and appropriately interpreted the interviewee’s consultations. Furthermore, the interviewer had received training in interviewing from a qualitative research expert beforehand. Both the interviewee (HS) and the interviewer (AA) were members of the authors.

#### Development and Verification of a Care Algorithm

##### Development of a Care Selection Algorithm Based on a Review of the Base Algorithm

Semistructured interviews were conducted with the interviewee. The interviewee reviewed the lists, sublists, and flowcharts of the base algorithm and reported any points she considered inappropriate for PI management in older patients at home. The interviewer modified the base algorithm according to the interviewee’s remarks. The interviewer continued the interview until there were no unclear points in the interviewee’s comments.

##### Test and Modification of the Care Algorithm Using Clinical Vignettes

The purpose of this process was to test how well the high-expertise algorithm developed in the previous process reflected the expertise of the interviewee, using PI clinical vignettes including PI photographs. First, the information of the case of PIs was input into the base and high-expertise algorithms. Both algorithms output care recommendations in 9 fields: (1) pressure relief; (2) nutritional management; (3) shear relief; (4) moisture management; (5) wound dressing use; (6) care for physical factors including bone prominence, obesity, joint contractures, and periwound edema; (7) care for systemic disorder; (8) selection of wound dressings, ointments, and negative pressure wound therapy; and (9) wound cleansing. These 9 fields were selected based on expert opinion. For instance, “Pressure relief” was included because PIs arise from sustained pressure, making this the most critical aspect of PI management. Likewise, “Moisture management” was incorporated because the skin becomes fragile under conditions such as incontinence, leading to maceration, which can significantly affect PI healing. The interviewee compared both outputs and evaluated the recommendations by the high-expertise algorithm on a 5-point scale: (0) both base and high-expertise algorithms perfectly agree with one’s thoughts, (1) worse than the base algorithm, (2) as bad as the base algorithm, (3) better than the base algorithm, and (4) better than the base algorithm and perfectly agrees with one’s thought. This procedure was performed in multiple vignettes, and the researcher (AA) calculated the agreement and improvement proportions. We treated that high similarity between the algorithms and the expert’s thoughts indicated the good performance of the algorithm because this study aimed to develop a care algorithm reflecting the expert thoughts.

The agreement proportion was defined as (the number of vignettes where score=0, 3, 4 or the total number of vignettes), and a higher number indicates better clinical applicability of the high-expertise algorithm. The improvement proportion was defined as (number of vignettes where score=3, 4 or number of vignettes where score=1, 2, 3, 4) and indicated the closeness of the high-expertise algorithm to the interviewee’s thoughts. If the score was 0 for all vignettes in 1 field, the improvement proportion of the field was defined as “NULL.” The evaluations were conducted in 3 rounds. After rounds 1 and 2, the interviewer asked about the disagreement between the high-expertise algorithm and the interviewee’s thoughts. The high-expertise algorithm was modified again based on her comments.

Finally, the interviewee rated the level of expected healing when all recommendations from the high-expertise algorithm were applied to the PI on a 4-point scale: (1) worsens, (2) stagnates, (3) improves, and (4) much improves. The score was rated once per vignette.

##### Details of Clinical Vignettes

The clinical vignettes for the test were devised by researchers based on records of the first assessment in which the interviewee provided consultations with nurses for PI management. In these records, patients were in home care or immediately after hospitalization, and their detailed records were available. If there were multiple PIs in 1 patient, the wound with the highest severity was used for the clinical vignettes. These records were collected when home-visiting nurses consulted the WOC nurse for PIs that did not show healing progress despite >2 weeks of management by home-visiting nurses. Cases in which a patient or their family expressed withdrawal of consent, PI was at the lower extremity, or there was no record to determine information were excluded. The vignettes were randomly assigned so that each round had the same number of cases.

### Study II

#### Outline

The authors determined predictors that are effective in estimating a predictive distribution of changes in the DESIGN-R 2020 score between assessments when an expert provides consultations [22,23]. Additionally, we developed a predictive model based on the selected predictors. DESIGN-R 2020 is a tool for evaluating PIs based on 7 parameters: depth, exudate, size, inflammation or infection, granulation tissue, necrotic tissue, and pocket size. The DESIGN-R 2020 score shows the sum of the 6 items, excluding depth. A higher score indicated a more severe PI with a longer healing time.

#### Data Source

Among records of the assessments in which the interviewee provided consultations with home-visiting nurses for PI management in a previous study [10], information on patients with d2 or deeper PIs was used in Study II. Multiple PIs in 1 patient were included as different PIs. Cases in which a patient or their family expressed withdrawal of consent, PI was in the lower extremity, consultations were conducted only once, and there was no record to judge predictors were excluded. The data were split into training and test data at a ratio of 80:20. To demonstrate the time robustness of the prediction model, 20% of the cases with longer follow-up periods were selected as test data.

#### Determining Predictors in Hierarchical Bayesian Model

##### Method Choice

Considering that the object is distribution estimation and the effect of predictors can vary for each individual, a hierarchical Bayesian regression model (HBR) was selected. The HBR is a method used to explain the distribution of a target variable assuming that the effect of predictors follows different distributions for each individual. Unlike generalized linear mixed models, HBR can estimate a posterior predictive distribution of target variables for which normality cannot be assumed, such as changes in DESIGN-R 2020 score. We used the HBR because it accounts for variability at both the patient and lesion levels and allows incorporation of prior knowledge, providing robust estimation even with limited data. By explicitly modeling uncertainty and hierarchical structure, the approach enables patient-specific predictions and can be updated as new data become available. The Markov chain Monte Carlo (MCMC) with the No U-Turn Sampler [28] was used to estimate the parameters. MCMC sampling was performed using the C++ compiler in Stan [29] and R [30]. The number of chains was set to 3, and 40,000 sampling iterations were performed after 10,000 warmups from the marginal posterior distribution per chain. Convergence of the MCMC samples to a stationary distribution was determined by the Gelman-Rubin R-hat statistic [31] and visually checking the trace plots.

##### Predictors

The predictors were as follows: the number of days from the latest assessment to the next assessment (DurationForNext) and its squared value, the number of days from the first assessment to the latest assessment (DurationFromDay0) and its squared value, DESIGN-R 2020 score at the first assessment and latest assessment, DESIGN-R 2020 subscale at the latest assessment, PI risk assessment factor items [32] (ability to change posture on bed, ability to maintain posture in chair, abnormal bony prominence, joint contracture, malnutrition, skin wetness, skin fragility [edema or skin tear]), and comorbidities (dementia, diabetes, liver disease, cardiovascular disease, cerebrovascular disease, respiratory disease, and cancer). Details of care were not used because there were no records to precisely determine them. Since the DESIGN-R 2020 subscales are classified as “Large” (severe) and “Small” (mild) based on their scores [22,23], the predictors were binarized as dummy variables according to the classification. For example, if the E score was 6, it was converted to “large” E, and if the E score was 1 or 3, it was converted to “small” e. When the E score was 0, both dummy variables were treated as 0.

##### Model Design

Let DESIGN-R 2020 score for a PI i at day t be $y_{i}^{\left(t\right)}$, and the changes in the DESIGN-R 2020 score after s days from day t be$d_{i}^{\left(t,s\right)}$. Then, we designed the HBR as follows:


$$d_{i}^{\left(t,s\right)}=y_{i}^{\left(t+s\right)}-y_{i}^{\left(t\right)}=f\left(X_{i}^{\left(t,s\right)}\right)$$

$$f\left(X_{i}^{\left(t,s\right)}\right)=X_{i}^{\left(t,s\right)}\beta_{i}+\epsilon_{i}^{\left(t,s\right)}$$

$$\epsilon_{i}^{\left(t,s\right)}∼Normal(0,\sigma^{2})$$

$$\left[\begin{array}{c} \begin{array}{cccccc} 1 & s & s^{2} & x_{1}^{\left(t\right)} & \cdots & x_{k}^{\left(t\right)} \end{array} \end{array}\right]$$

$$\beta_{i}∼MultiNormal(\vec{\mu_{\beta }},\Sigma_{Chol})$$

$$\vec{\mu_{\beta }}={\left[\mu_{intercept},\mu_{\beta_{s},}\mu_{\beta_{x_{1}},},\cdots \mu_{\beta_{x_{k}},}\right]}^{T}or$$

$${\left[\mu_{intercept},\mu_{\beta_{s},}\mu_{\beta_{s^{2}}}\mu_{\beta_{x_{1}},},\cdots \mu_{\beta_{x_{k}},}\right]}^{T}$$

where

$X_{i}^{\left(t,s\right)}$ is a vector of predictors at day t.

$\beta_{i}$ is a parameter vector depending on PI i.

$\epsilon_{i}^{\left(t,s\right)}$is an error term following a normal distribution with mean 0 and variance $\sigma^{2}$.

$\vec{\mu_{\beta }}$ is a hierarchical mean parameter vector of a multivariate normal distribution $\beta_{i}$ follows. $\vec{\mu_{\beta }}$ expresses the fixed effects of predictors.

$\Sigma_{Chol}$ is a Cholesky factor of the variance-covariance matrix of a multivariate normal distribution $\beta_{i}$ follows.

Since $d_{i}^{\left(t,s\right)}$ was often negative, logarithmic transformation for $d_{i}^{\left(t,s\right)}$ was not conducted. The prior distributions of the parameters were set as follows:


$$\sigma ∼Normal^{+}(0,100)$$

$$\mu_{\beta_{s},}\mu_{\beta_{s^{2}}}\mu_{\beta_{x_{1}},},\cdots \mu_{\beta_{x_{k}},}∼Normal(0,1)$$

$$\mu_{intercept}∼Normal(0,10)$$

$$\Sigma_{chol}=diag\left(\vec{\zeta_{\beta }}\right)\cdot \Omega_{Chol}$$

$$\vec{\zeta_{\beta }}=\;\zeta_{Intercept},\;\zeta_{\beta_{s}},\zeta_{\beta_{s^{2}}},\zeta_{\beta_{1}},\;\cdots \;\zeta_{\beta_{k}}∼Student\_t^{+}\left(4,\;0,\;1\right)$$

$$\Omega_{Chol}∼LkjCorr(4)$$

where

$\vec{\zeta_{\beta }}$ is a hierarchical variance parameter vector of $\beta_{i}$. $\vec{\zeta_{\beta }}$ expresses the random effects of predictors.

$\Omega_{chol}$ is a Cholesky factor of the correlation matrix that follows the Lewandowski-Kurowicka-Joe distribution [33] with a shape parameter of 4.

##### Predictor Selection

Predictors that improve the fitness of the data when added to the base model were determined in the training data. The base model was set as a model that predicts $d_{i}^{\left(t,s\right)}$with only s, that is, DurationForNext. Then, the base model was set to $M_{0}$, and the model with s and another predictor p was set to $M_{p}$. Subsequently, the posterior probabilities [34] of $M_{p}$ for $M_{0}$ were calculated for all p under the assumption that the prior probabilities of $M_{p}$ and $M_{0}$ are equal. Posterior probability represents the probability that $M_{p}$ is closer to the true model compared to $M_{0}$. We selected all predictors whose posterior probability was >0.95 and developed a predictive model with all combinations of the selected predictors. The combination with the highest log marginal likelihood was regarded as the candidate for the best model. After 5 repetitions of determining a candidate of the best model in 80% of the randomly separated training data, log marginal likelihoods were calculated for the candidates in all training data. The model with the highest log marginal likelihood in the 5 candidates was determined to be the best model. The log marginal likelihoods were the mean value of 5 approximations computed by bridge sampling using the Warp-III method [35] with R package “bridgesampling” [36].

### Validation of the Best Model Using Test Data

The changes in the total DESIGN-R 2020 score from each assessment to the next assessment were predicted using the test data with the best model. $\vec{\zeta_{\beta }}$ at the first prediction was set to 0. We calculated a coverage probability of 90% prediction interval to verify the performance of the predictive model. The coverage probability of a 90% prediction interval was defined as follows: the number of times when actual scores were within the 90% prediction interval or the number of assessments when the prediction was conducted. If this value is close to 0.9, it supports the possibility that the best model can estimate the changes in DESIGN-R 2020 score.

### Ethical Considerations

This study was approved by the Ethics Committee of the Graduate School of Medicine, the University of Tokyo (No. 2021277NI). All patients were provided with the opportunity to opt out of the study. All data were handled in a manner that ensured participants could not be individually identified. No honoraria or other forms of compensation were provided for participation in this study.

## Results

### Study I

**Figure 2.**
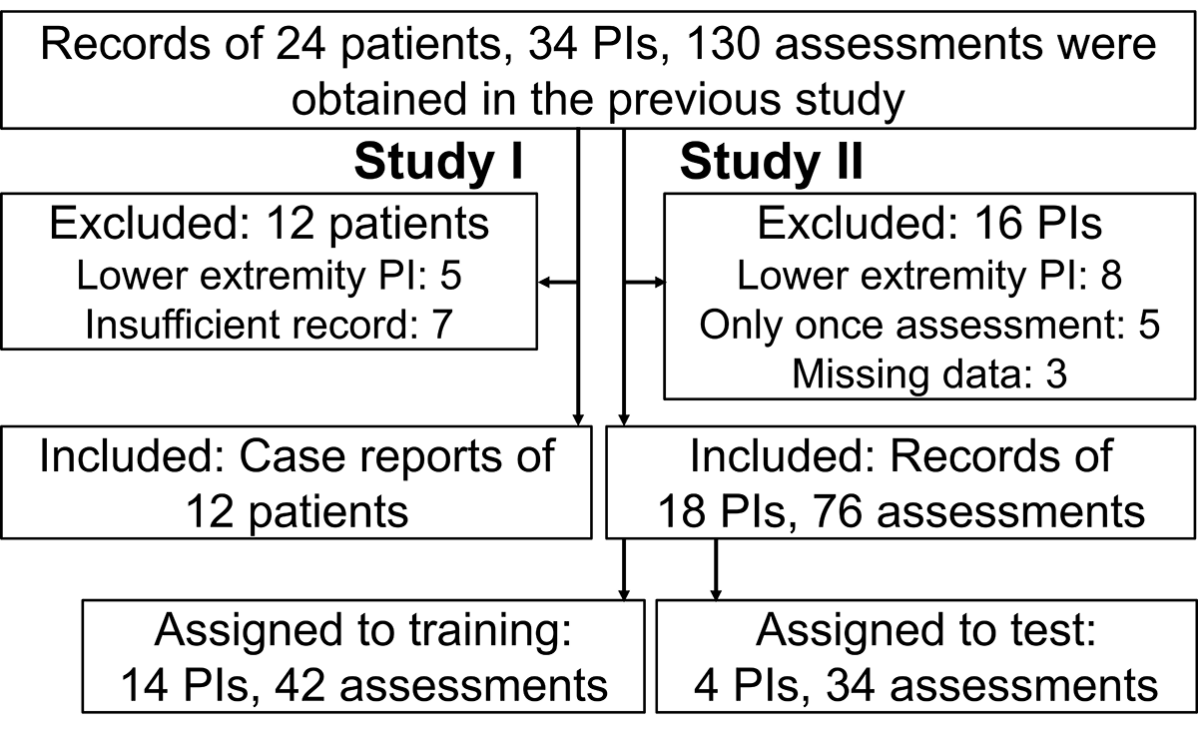
Flowchart showing process of inclusion. PI: pressure injury.

**Figure 3.**
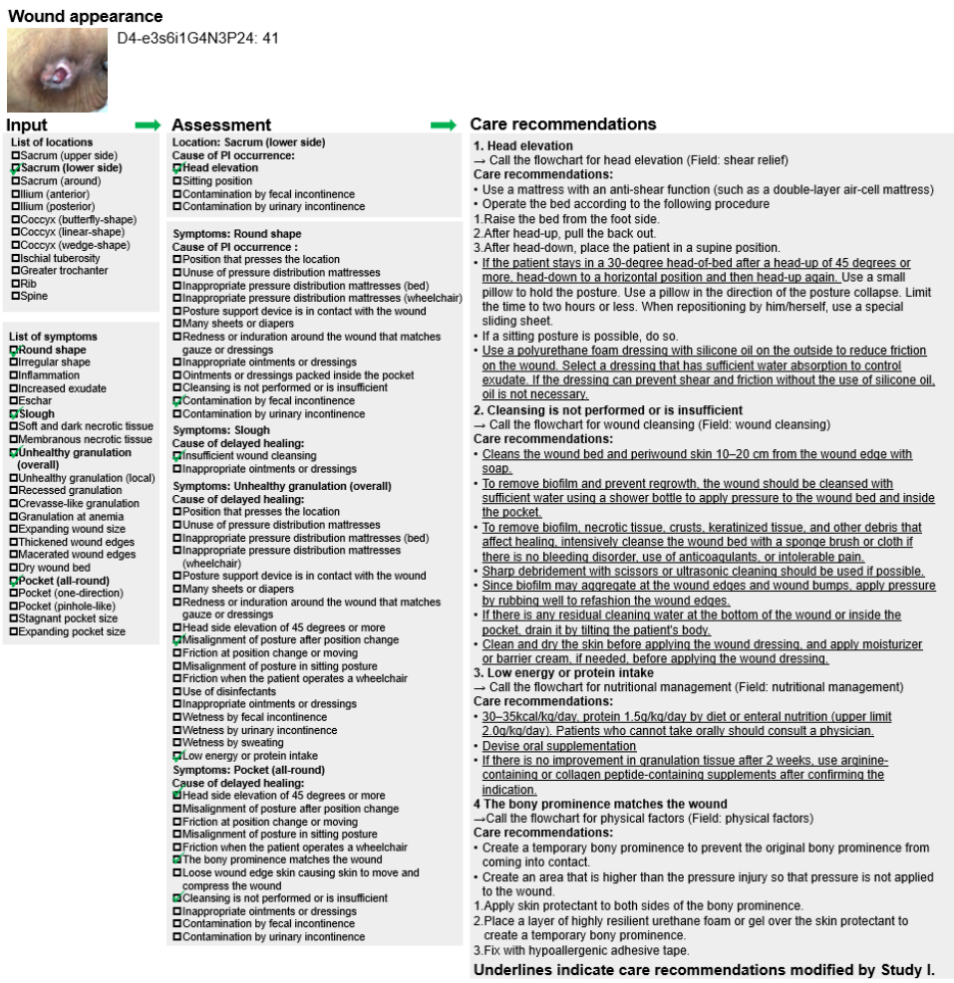
Examples of care recommendation outputs.

A total of 12 clinical vignettes were used in this study (Figure 2). Figure 3 represents a detailed example of care recommendations based on the high-expertise algorithm. The sample size was 4 in all rounds. The results of the high-expertise algorithm test are listed in (Tables 1 and 2).

The means of agreement proportion were 0.92 (33/36), 0.75 (27/36), and 0.89 (32/36) for each round, and the means of improvement proportion were 0.73 (8/11), 0.25 (3/12), and 0.76 (13/17), respectively. The mean expected healing scores were 2.67, 3.00, and 3.25, respectively.

**Table 1.** Result of the test of the modified algorithm with clinical vignettes: the agreement proportions.

Fields	Round 1 (n=4)	Round 2 (n=4)	Round 3 (n=4)
Pressure relief	0.75 (3/4)	1.00 (4/4)	1.00 (4/4)
Nutritional management	1.00 (4/4)	1.00 (4/4)	0.75 (3/4)
Shear relief	1.00 (4/4)	0.75 (3/4)	1.00 (4/4)
Moisture management	0.75 (3/4)	1.00 (4/4)	0.75 (3/4)
Wound dressing use	1.00 (4/4)	1.00 (4/4)	1.00 (4/4)
Care for physical factor	1.00 (4/4)	1.00 (4/4)	1.00 (4/4)
Care for systemic disorder	0.75 (3/4)	0.75 (3/4)	0.75 (3/4)
Selection of wound dressings, ointments, and negative pressure wound therapy	1.00 (4/4)	0.25 (1/4)	0.75 (3/4)
Wound cleansing	1.00 (4/4)	0.00 (0/4)	1.00 (4/4)
Total	0.92 (33/36)	0.75 (27/36)	0.89 (32/36)

**Table 2.** Result of the test of the modified algorithm with clinical vignettes: the improvement proportions.

Fields	Round 1 (n=4)	Round 2 (n=4)	Round 3 (n=4)
Pressure relief	0.50 (1/2)	NULL	NULL
Nutritional management	1.00 (1/1)	1.00 (1/1)	0.67 (2/3)
Shear relief	NULL	0.50 (1/2)	1.00 (3/3)
Moisture management	0.50 (1/2)	NULL	0.5 (1/2)
Wound dressing use	NULL	1.00 (1/1)	NULL
Care for physical factor	1.00 (1/1)	NULL	NULL
Care for systemic disorder	0.00 (0/1)	0.00 (0/1)	0.50 (1/2)
Selection of wound dressings, ointments, and negative pressure wound therapy	1.00 (3/3)	0.00 (0/3)	0.75 (3/4)
Wound cleansing	1.00 (1/1)	0.00 (0/4)	1.00 (3/3)
Total	0.73 (8/11)	0.25 (3/12)	0.76 (13/17)

### Study II

#### Characteristics and DESIGN-R 2020 Score Changes

A total of 76 assessment records from 18 PIs were included in the eligible data (Figure 2). A total of 42 records from 14 PIs were assigned to the training data, and 34 records from 4 PIs were assigned to the test data. The characteristics of the patients and PIs are presented in Table 3.

**Table 3.** Characteristics of pressure injuries included in Study II.

Characteristics	Training (n=14)	Test (n=4)
Days of follow-up, mean (SD)	29.4 (17.3)	81.2 (23.3)
Age (y)	79.4 (8.4)	85.0 (9.3)
Sex (female), n (%)	6 (43)	4 (100)
Pressure injury location, n (%)		
Sacrum	5 (36)	3 (75)
Coccyx	3 (21)	1 (25)
Others	6 (43)	0 (0)
Pressure injury risk factor, n (%)		
Ability to change posture on bed	9 (64)	3 (75)
Ability to maintain posture in chair	9 (64)	3 (75)
Abnormal bony prominence	6 (43)	1 (25)
Joint contracture	5 (36)	1 (25)
Malnutrition	8 (57)	2 (50)
Wet skin	6 (43)	1 (25)
Vulnerable skin (edema)	4 (29)	2 (50)
Vulnerable skin (skin tear)	6 (43)	1 (25)
Disease, n (%)		
Dementia	5 (36)	3 (75)
Diabetes	3 (21)	0 (0)
Liver disease	2 (14)	0 (0)
Cardiovascular disease	2 (14)	2 (50)
Cerebrovascular disease	5 (36)	1 (25)
Respiratory disease	2 (14)	0 (0)
Cancer	2 (14)	0 (0)

**Figure 4.**
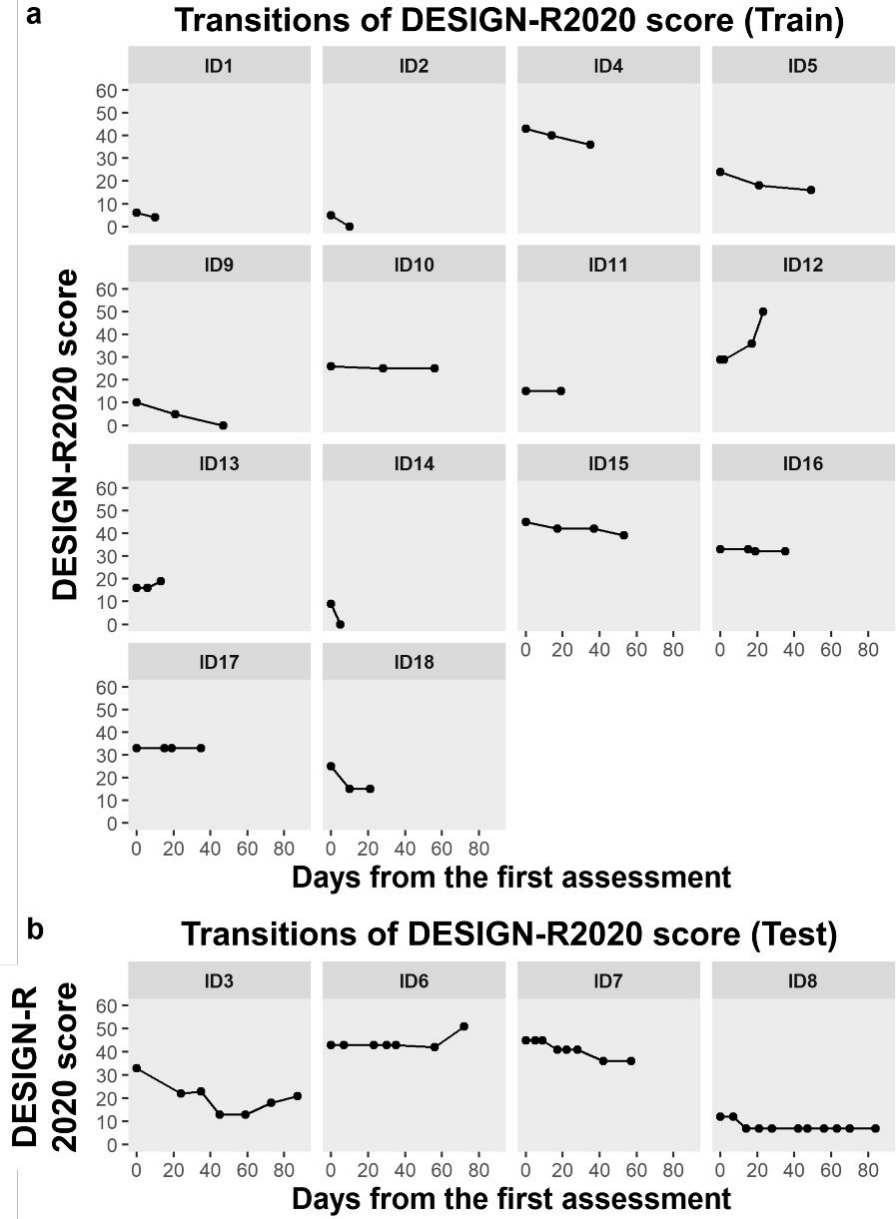
Transitions of DESIGN-R 2020 score.

The mean follow-up periods were 29.4 (SD 17.3) days and 81.2 (SD 23.3) days for the training and test data, respectively. Changes in the DESIGN-R 2020 scores for each case are shown in Figure 4.

#### Determination of the Best Model

The predictors included in the best model and its candidates developed with the training data, and the log marginal likelihoods of the model are shown in Table 4.

**Table 4.** Selected predictors and log marginal likelihood in the best model.

Dataset^a^	Predictors included in the best model in each dataset	Log marginal likelihood
Fold 1	Age, DurationForNext, (DurationForNext)^2^, (DurationFromDay0)^2^	96.143
Fold 2	Age, DurationForNext, (DurationForNext)^2^, (DurationFromDay0)^2^	103.128
Fold 3	Age, DurationForNext, (DurationForNext)^2^, DurationFromDay0, (DurationFromDay0)^2^, DESIGN-R2020 score at the first assessment	110.225
Fold 4	Age, DurationForNext, (DurationForNext)^2^, (DurationFromDay0)^2^, DESIGN-R2020 score at the first assessment	100.325
Fold 5	Age, DurationForNext, (DurationForNext)^2^, (DurationFromDay0)^2^, DESIGN-R2020 score at the first assessment	110.698
All training data	Age, DurationForNext, (DurationForNext)^2^, DurationFromDay0, (DurationFromDay0)^2^, DESIGN-R2020 score at the first assessment	116.874

a Each fold was composed of 80% of the data randomly selected from all the training data.

The best model included age, DurationForNext and its squared value, DurationFromDay0 and its squared value, and DESIGN-R 2020 at the first assessment scores. Predictors in the candidates of the best model in each fold did not completely match, whereas all candidates included age and (DurationForNext)^2^ in addition to DurationForNext. The Gelman-Rubin R-hat statistics of all parameters in the best model were less than 1.1, and the traceplot indicated that the MCMC samples were well mixed. A summary of marginal posterior distributions of σ, $\vec{\mu_{\beta }}$, $\vec{\zeta_{\beta }}$ of the best model is shown in Table 5. The posterior mean, which is the mean of samples from marginal posterior distributions, of the hierarchical mean parameter of age and DESIGN-R 2020 at the first assessment score was 0.05 and −0.10, respectively.

**Table 5.** Summary of marginal posterior distribution of hierarchical parameters in the best model^a^.

Parameter	Mean parameter (μ^b^), mean (SD)	Variance parameter (ζ^b^), mean (SD)
DurationForNext	−0.55 (0.44)	0.17 (0.12)
(DurationForNext)^2^	0.02 (0.02)	0.01 (0.01)
DurationFromDay0	−0.52 (0.22)	0.26 (0.15)
(DurationFromDay0)^2^	0.02 (0.01)	0.01, (0.01)
Age (y)	0.05 (0.11)	0.04 (0.02)
DESIGN-R 2020 score at the first assessment	−0.10 (0.13)	0.13 (0.09)
Intercept	3.91 (8.11)	0.97 (0.83)

a DurationForNext; the number of days from the latest assessment to the next assessment, DurationFromDay0; the number of days from the first assessment to the latest assessment.

b The expected a posterior estimation and its standard deviation of samples.

#### Prediction With the Best Model

**Figure 5.**
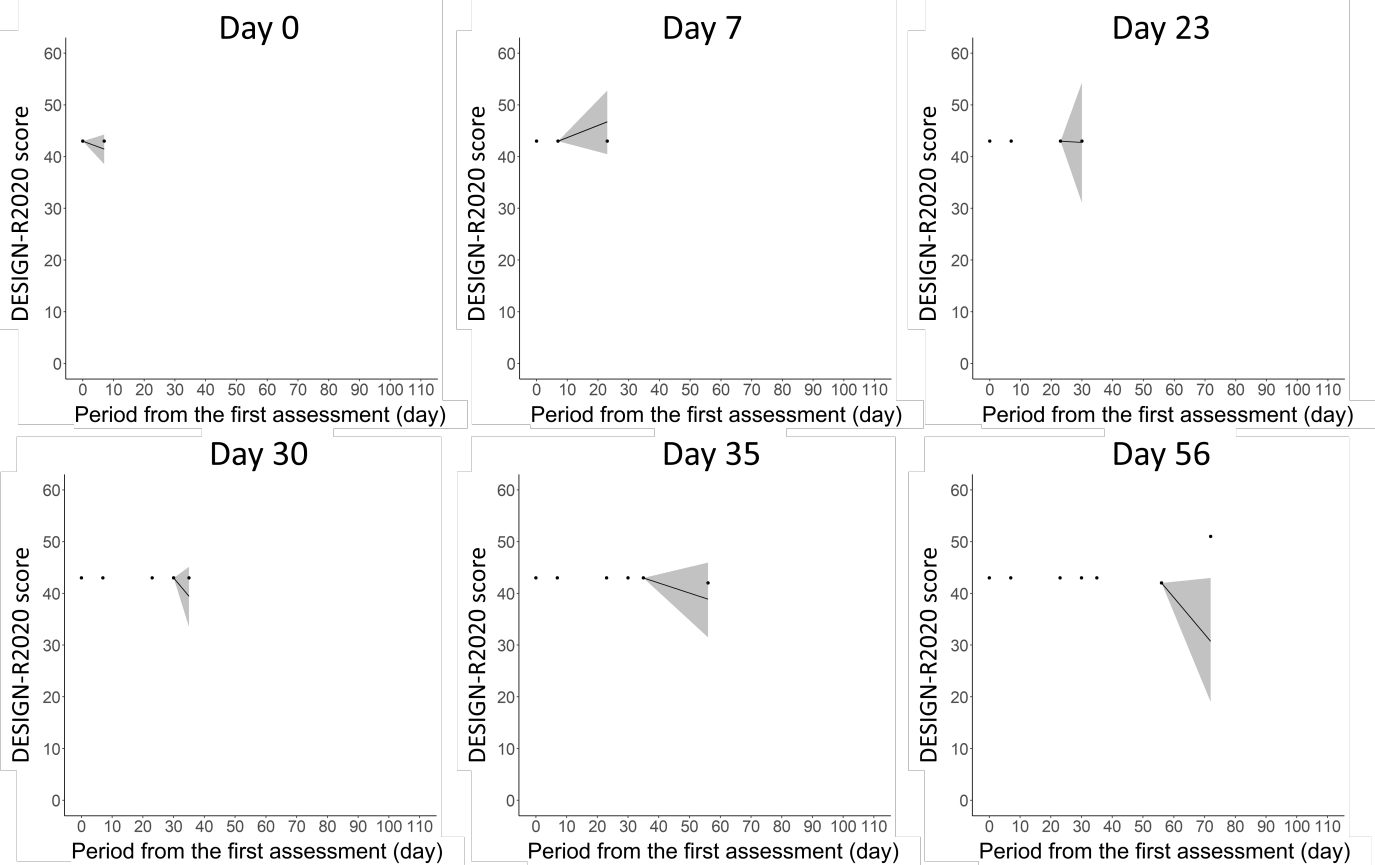
An example of a 90% prediction interval for 1 patient in test data calculated in the best model.

Coverage probabilities of the 90% prediction interval among the 4 PIs in the test data were 0.67 (4/6), 0.83 (5/6), 0.86 (6/7), and 0.80 (8/10), respectively. The mean of these coverage probabilities was 0.79 (23/29). An example of the comparison between the actual DESIGN-R 2020 scores and that of 90% predicted intervals in the test data is shown in Figure 5. In this patient, the predictions made from Day 0 to Day 35 included the score at the next assessment in the 90% prediction interval, and the 90% prediction interval for Day 56 did not include the Day 72 score. The worsening of the infection at the time of the last assessment (Day 72) resulted in a 9-point increase in the DESIGN-R 2020 score, which was a rare case under expert nurse consultation.

## Discussion

### Principal Results

The goal is to develop a CDSS that guides home-visiting nurses on appropriate care based on a high-expertise algorithm reflecting a thought of a PI expert and a predictive model that estimates the predictive distribution of the changes in DESIGN-R 2020 to inform nurses whether consultation from an actual expert nurse is needed. This unique idea can develop a CDSS that provides more appropriate care recommendations for patients who require expert wound management in home care settings. Study I developed an algorithm by modifying an existing algorithm with the interviewee’s expertise. In Study II, the predictive distribution of changes in the DESIGN-R 2020 score can be reasonably estimated using the patient’s age and DESIGN-R 2020 score at the first assessment, and the prediction model was successfully developed.

In Study I, the test of the high-expertise algorithm was designed based on the CDSS validation procedure presented by Rodríguez-González et al [37]. In this study, the referee was the interviewee, and the interviewer prepared the information for the test cases. The referee then judged the closeness of the outputs of the base and high-expertise algorithms to her judgment. Since algorithms should also be assessed quantitatively [38], the closeness and the level of expected healing in the high-expertise algorithm were investigated quantitatively. Furthermore, the test could be regarded as a member check from a qualitative research perspective. Since the base algorithm was developed in 2003, the content of modifications included not only the unique expertise of the interviewee but also the present clinical knowledge. However, we did not distinguish between expertise and evidence because expertise includes the evaluation of evidence and new knowledge [39]. Based on these efforts, the process of algorithm modification was considered internally valid.

In Study I, the agreement and improvement proportions were lower in Round 2 compared with Rounds 1 and 3, particularly for “(8) selection of wound dressings, ointments, and negative pressure wound therapy” and “(9) wound cleansing.” Wound dressings and wound cleansing were areas in which the high-expertise algorithm introduced many modifications compared with the base algorithm. In Round 1, relatively few PIs had wound characteristics relevant to these items, whereas in Round 2, many PIs with such characteristics were included, which likely resulted in lower proportions. After further refinements following Round 2, the algorithm was able to provide appropriate recommendations even for PIs with these characteristics in Round 3. The results in Study I showed that the recommendations of the high-expertise algorithm were 75%‐92% consistent with the interviewee’s thoughts. Since generally guidelines are applicable to only about 60%‐95% of patients [40], the agreement proportions indicate the high clinical applicability of the developed algorithm. On the other hand, the improvement proportion was not stable. For example, the low improvement proportion for nutritional management indicates that the care recommendations related to nutrition did not contribute substantially to PI improvement. In home care settings, it is often difficult to obtain detailed nutritional information or to modify patients’ diets. The system can be further improved by adding more cases. Also, we excluded vignettes where both the base and high-expertise algorithms provided appropriate recommendations when calculating improvement proportions because it allowed us to focus on clinically meaningful changes rather than diluting the effect with unchanged cases. However, excluding the vignettes may have slightly inflated the observed mean improvement. In addition, the high expected healing level of 3.25 on a 4-point scale suggests that the algorithm developed in this study has the potential to contribute to PI improvement. By referring to care recommendations based on this algorithm, nurses may be able to provide more effective PI management compared to assessments and care decisions made solely by nurses.

In Study II, we created a model to predict changes in the DESIGN-R 2020 score, while previous studies have predicted a change of wound area or length of wound edge. Although the DESIGN-R 2020 score is the sum of the integer values of 6 subscales making the changes discrete, it includes the pathophysiology of PIs such as infection and necrotic tissue. Nurses select appropriate care based not only on wound size but also on the pathophysiology of PIs, such as infection and necrotic tissue. Because the DESIGN-R2020 score reflects both of these aspects, using changes in this score as the target variable is reasonable. The sampling was appropriate because MCMC samples from the best model were converged to a stationary distribution and the Gelman-Rubin R-hat statistics of all parameters were less than 1.1, indicating adequate convergence.

In addition to predictors related to time, DESIGN-R 2020 score at the first assessment and the patient’s age were included in the best model. It is noteworthy that the hierarchical mean parameter of age in the best model showed that the PIs of older patients were more likely to heal slightly sooner. As all the PIs in this study were hard to heal, it is possible that younger patients had some barrier in their general condition, or the care environment was not comparable to that of the older patients. The posterior mean of the hierarchical mean parameter of DESIGN-R 2020 score at the first assessment in the best model was negative. This may suggest that a PI diagnosed as severe at first assessment may take longer to heal, even if the causes of delayed healing were resolved by expert consultation. Unlike in a previous study [18], the location of the PI was not included, because all the PIs in this study were in the trunk or greater trochanter and not in the lower extremities. The best model did not include predictors related to comorbidities or PI risk assessment factor items. These factors may not have affected the healing progress, as they were appropriately addressed by expert nurse consultation. The mean coverage probability of 90% prediction interval was 0.79, which indicates that the model underestimated uncertainty. It means that predicted intervals are narrower than they should be. While this indicates that the best model still has room for improvement, this may adequately estimate the distribution of changes in DESIGN-R 2020 scores at a clinically acceptable level for a longer range than a duration of training data. Other predictors, such as the cause of PI development and delayed healing, and materials used in PI care may improve the performance of the model, allowing for more patient-specific predictions. Also, accessibility to health care resources could also be considered because health care delivery is sometimes difficult in rural areas.

The incorporation of nurse expertise through interviews could also be applied to other chronic disease management domains, such as diabetes and heart failure, where recommendations may support care decisions related to patient self-care education, symptom management, and reporting of abnormalities to physicians. An ethical challenge includes the protection of patient privacy, while a practical challenge may arise from the greater diversity of lifestyle factors compared with PI management, which could make it more difficult to develop a highly accurate CDSS.

### Limitations

The small sample size and low variety of PIs may limit both performance and generalizability of the algorithm and the predictive model. Since the expected healing level is determined by 1 expert in Study I, it is not clear how other experts assess the expected healing level for this care algorithm. In addition, we did not split the records from the same PIs into training and test data because they involved the same patient characteristics, and care recommendations vary not only according to the condition of the PIs but also depending on patient characteristics. This may have led the algorithm to perform well for patients with similar conditions.

Study II employed comorbidities reported by home-visiting nurses as predictors that reflect the general conditions indirectly. However, it may have included diseases already well controlled to a level that did not affect the general condition. Hence, the effect of the general condition on PI healing may have been underestimated, and using predictors that directly represent the general condition, such as HbA_1c_ and SpO_2_, may improve the model.

Future research should include a prospective evaluation of the high-expertise algorithm in real-world clinical settings. In particular, the effectiveness of the algorithm could be assessed through the monitoring of DESIGN-R 2020 scores, while safety should be evaluated using indicators such as hospitalizations due to PI worsening and the incidence of PI infections. Such a study would provide important evidence regarding the ongoing clinical utility and safety of the algorithm and inform further refinements for broader implementation.

### Conclusions

This study was conducted as the first step in the development of a CDSS for home-visiting nurses in PI management. We developed an algorithm that reflected the expert’s knowledge in the existing algorithm and showed that the high-expertise algorithm was clinically applicable and close to the wound expert’s thoughts. It also determined predictors effective for predicting changes in DESIGN-R 2020 score of PI in home care settings and developed a model to calculate the prediction distribution. This enables the CDSS to inform nurses that it cannot address the patient’s management appropriately as a fail-safe mechanism.

## Abbreviations

CDSS: clinical decision support systems
HBR: hierarchical Bayesian regression model
MCMC: Markov chain Monte Carlo
PI: pressure injury
WOC: wound, ostomy, and continence
